# CCProf: exploring conformational change profile of proteins

**DOI:** 10.1093/database/baw029

**Published:** 2016-03-25

**Authors:** Che-Wei Chang, Chai-Wei Chou, Darby Tien-Hao Chang

**Affiliations:** Department of Electrical Engineering, National Cheng Kung University, Tainan, 70101, Taiwan

## Abstract

In many biological processes, proteins have important interactions with various molecules such as proteins, ions or ligands. Many proteins undergo conformational changes upon these interactions, where regions with large conformational changes are critical to the interactions. This work presents the CCProf platform, which provides conformational changes of entire proteins, named conformational change profile (CCP) in the context. CCProf aims to be a platform where users can study potential causes of novel conformational changes. It provides 10 biological features, including conformational change, potential binding target site, secondary structure, conservation, disorder propensity, hydropathy propensity, sequence domain, structural domain, phosphorylation site and catalytic site. All these information are integrated into a well-aligned view, so that researchers can capture important relevance between different biological features visually. The CCProf contains 986 187 protein structure pairs for 3123 proteins. In addition, CCProf provides a 3D view in which users can see the protein structures before and after conformational changes as well as binding targets that induce conformational changes. All information (e.g. CCP, binding targets and protein structures) shown in CCProf, including intermediate data are available for download to expedite further analyses.

**Database URL**: http://zoro.ee.ncku.edu.tw/ccprof/

## Introduction

Conformational changes are commonly observed in various protein interactions (1). For example, adenylate kinase, which catalyzes the phosphoryl transfer from adenosine triphosphate to adenosine monophosphate, undergoes a large conformational variation from an ‘open’ state to a ‘closed’ state (2). These conformational changes can be linked to many biological processes, such as substrate/ligand binding (3), protein–protein recognition (4), transcriptional regulation (5) and post-translational modifications like phosphorylation (6). Protein regions with large conformational changes are observed to have some biological patterns, such as having secondary structure changes (7), undergoing disorder to order transitions (7) and being highly conserved (8). Understanding protein conformational changes and their causes helps to study related biological functions.

To date, several related databases have been proposed. The MolMovDB (9) provides the animations of conformational changes. However, using MolMovDB to quantify conformational changes and to study the causes of conformational changes is difficult. The ComSin (10) database, which is designed for studying intrinsic protein disorders, provides protein structures in bound (complex) and unbound (single) states. The AH-DB (11) is another protein structure pair database, which contains >700 000 entries. The PCDB (12) is a domain database designed for studying conformational diversity, but it has been unavailable for a while. The CoDNaS (13) is another conformational diversity database, which contains >9000 proteins with >263 000 conformers. Compared with ComSin and AH-DB, PCDB and CoDNaS provide protein structure clusters rather than pairs. Among above databases, AH-DB and CoDNaS contain the most entries and are extensively annotated (taxonomy, protein function, ligands, etc.). The above databases provide valuable but primitive data for protein structure pairs/clusters. Although the data can be used to derive various information such as conformational change, users have to perform the calculation on their own. Furthermore, these databases use a global index (root-mean-square deviation, RMSD), to indicate conformational change. However, some conformational changes occur locally, such as those induced by ligand binding. In this regard, Protein Structural Change DataBase (PSCDB) (14) provides quantified conformational changes for 685 proteins at only regions but only for those with known causes. Namely, PSCDB is suitable for studying known conformational changes rather than elucidating novel ones.

Information visualization is another important issue for studying conformational change. In many cases, important observations can only be made when multiple datatypes are considered simultaneously. For example, to analyze the relationship between protein regions with large conformational changes and phosphorylation sites, one may prepare two lists of residues (one for protein regions with large conformational changes and the other for phosphorylation sites) and then conduct a list comparison algorithm. For researchers without a programming background, this procedure is difficult to perform.

This work presents the CCProf platform, which provides conformational changes of entire proteins, named conformational change profile (CCP) in the context. The CCP and the CCProf interface are designed to solve the above problems. Precisely, the purpose of CCProf is to provide users with a platform for studying potential causes of novel conformational changes in a wide range of analyses. To achieve this goal, providing conformational changes of entire proteins is necessary. For example, Bennett and Steitz (15) plotted a CCP to study the glucose-induced conformational change in yeast hexokinase. Dobbins *et al.* (16) use such a profile to analyze protein flexibility and interactions. Furthermore, CCProf provides 10 biological features, including conformational change, potential binding target site, secondary structure, conservation, disorder propensity, hydropathy propensity, sequence domain, structural domain, phosphorylation site and catalytic site for elucidating causes of conformational changes. Finally, all these information are compiled in a unified manner, named profile in the context, so that they can be aligned and presented simultaneously. This visual design is critical for researchers to capture important relevance between different biological features. The CCProf contains 986 187 protein structure pairs for 3123 proteins. All information (e.g. CCP, binding targets and protein structures) shown in CCProf, including intermediate data (e.g. sequence/structure alignments) are available for download. This is useful for conducting further analyses as well as for repeating experiments in other works.

## Materials and methods

Profiles shown in CCProf can be roughly classified into two categories based on how they are generated. The first category, which is generated by CCProf, contains four profiles: (i) conformational change, (ii) potential binding target site, (iii) secondary structure and (iv) conservation. In the four profiles, (i) is proposed in this work while (ii), (iii) and (iv) are calculated by CCProf based on commonly used definitions. The second category, which is obtained from public databases, contains six profiles obtained from public databases: disorder propensity, hydropathy propensity, sequence domain, structural domain, phosphorylation site and catalytic site.

## Data collection

The first step of calculating conformational changes is to collect protein structure pairs under different states. This work collects protein structure pairs before and after binding as well as the corresponding binding targets from the Protein Data Bank (PDB) database (17). This work defines the state of a protein structure in a PDB file according to whether it binds target molecules in that PDB file. Since one PDB file may contain multiple molecules in a complex structure, this section uses the term ‘structure’ to refer to the coordinates of a single biological unit in a PDB file. The procedure of pairing protein structures of the same protein under different states consists of three steps. First, PDB files of X-ray crystallographic biological units are downloaded. Second, two protein structures *s*_1_ and *s*_2_ are paired if three conditions hold: (i) *s*_1_ and *s*_2_ are in different PDB files (suppose that *s*_1_ in PDB file *F*_1_ and *s*_2_ in PDB file *F*_2_), (ii) *s*_1_ and *s*_2_ overlap and (iii) *F*_1_ contains all structures in *F*_2_ and at least one extra structure. The details of overlap detection are described in the next paragraph. Third, 10 biological profiles are generated for each structure pair.

**Figure 1.**
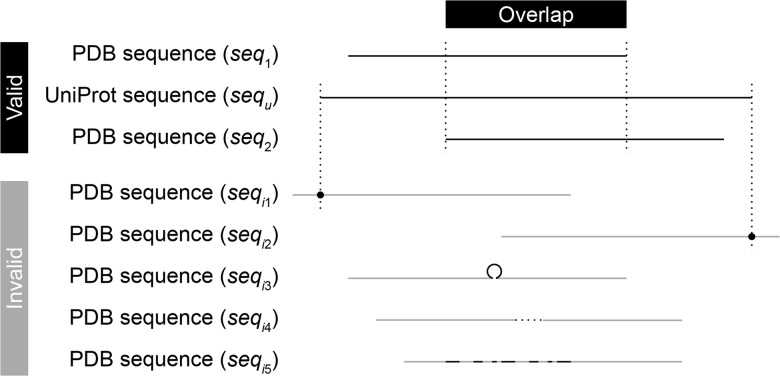
Overlap detection. To detect the overlap between two PDB sequences seq_1_ and seq_2_, CCProf conducts two sequence alignments to map them individually onto the corresponding UniProt sequence (seq_u_). In addition to requiring an overlap between the two mapped regions, the alignments of either sequences must not fall into any of the invalid cases. (seq_i1_), The alignment starts in the middle of the sequence; (seq_i2_), the alignment ends in the middle of the sequence; (seq_i3_), the sequence has an insertion in the alignment; (seq_i4_), the sequence has an deletion in the alignment and (seq_i5_), the sequence is not similar enough (identity < 95% or *e* value ≥ 0.001) against sequ.

In the second step of structure pairing, CCProf introduces a refined alignment scheme to determine whether two structure overlap. The scheme is used to overcome the challenge that PDB files may contain only protein fragments. Directly aligning two protein fragments may yield incorrect local alignments. In CCProf, the overlap of two protein structures *s*_1_ and *s*_2_ of the same protein *p* are determined via two sequence alignments. Structural alignments are performed later to calculate conformational changes and to generate superimpose structures (see Conformational change section). As shown in Figure 1, this work maps seq_1_ and seq_2_ onto seq*_u_* with the Basic Local Alignment Search Tool (18) to detect the overlap between *s*_1_ and *s*_2_. Sequences seq_1_ and seq_2_ are generated from the SEQRES records corresponding to *s*_1_ and *s*_2_, respectively; while seq*_u_* is obtained from the UniProt, which stands for the complete sequence of *p*. CCProf considers that *s*_1_ and *s*_2_ overlap if their alignments on *p* satisfy five conditions: (i) start and end at sequence ends (i.e. seq_1_ and seq_2_ are subsequences of seq*_u_*), (ii) have no insertion and deletion, (iii) identity ≥ 95%, (iv) *e* value < 0.001 and (v) overlap. The extra structure(s) in *F*_1_ against *F*_2_ within five angstroms to *s*_1_ are denoted ‘binding targets’.

The six profiles obtained from public databases are outlined here while the details of the four profiles generated by CCProf are described in the following subsections. The first profile is disorder propensity, which indicates the inverse propensity of each residue to have a stable structure (19). This profile is obtained from PDB. The second profile is hydropathy propensity, which shows the hydropathy sum of the proximity (15 residues) for each residue (20). This profile is obtained from PDB. The third profile is sequence domain, which is a conserved protein subsequence that can function independently (21). This profile is obtained from three databases: PDB site, UniProt motif (22) and Pfam domain (23). The UniProt is a comprehensive repository of protein sequences and annotation, whereas the Pfam is a large collection of sequence domain families. The fourth profile is structural domain, which is a frequent observed substructure that can fold independently. This profile is obtained from the Structural Classification of Proteins database (24). The SCOP is a database of structural classification for proteins. The fifth profile is phosphorylation site, which is a specific protein region that carries out addition or removal of a phosphate group and is critical to protein activation/deactivation (25). This profile is obtained from UniProt and the Phospho.ELM database (26). The Phospho.ELM stores *in vivo* and *in vitro* phosphorylation data extracted from literature and phosphoproteomic analyses. The sixth profile is catalytic site, which is a small region in enzymes to bind substrates and conduct chemical reactions. This profile is obtained from the Catalytic Sites Atlas database (27). The Catalytic Sites Atlas provides catalytic residues annotation for enzymes.

## Conformational change

**Figure 2.**
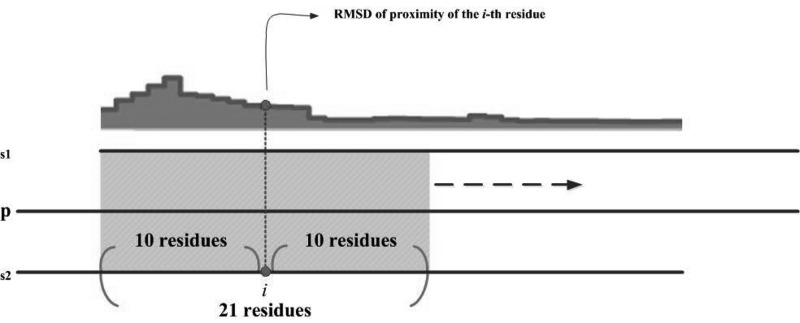
Schematic diagram of sliding local alignment. The sequences of seq_1_ and seq_2_ are generated from the SEQRES records in PDB files; the sequence of seq_u_ is obtained from the UniProt database. This work uses a sliding window of 21 residues (10 leading and 10 trailing of current position) to scan seq_u_. For each position, the value on the profile is the RMSD of structurally aligning the corresponding residues in seq_1_ and seq_2_. Only residues appearing in both seq_1_ and seq_2_ are considered.

A CCP in this work refers to a profile on which position *i* indicates the intensity of structural variation in the proximity of the *i*th residue of a protein. RMSD, a commonly used index in structural alignment (28,29), is used to measure the intensity of structural variation. As described in Data collection section, this work does not directly perform a structural alignment on *s*_1_ and *s*_2_ because that PDB files may contain only protein fragments. A UniProt sequence of the corresponding protein, *p*, is introduced to represent the entire protein as well as a ruler in CCProf. All profiles are mapped onto the UniProt sequence, seq*_u_*, so that CCProf can align and present them all together. After *s*_1_ and *s*_2_ are mapped on seq*_u_*, a sliding local structural alignment of 21 residues is carried out along seq*_u_*, and the resultant series of RMSDs form the CCP of *p* (Figure 2). Assume *r_u_*(*i*) is the *i*th residue in seq*_u_*; *r*_1_(*i*) and *r*_2_(*i*) are the residues mapped to *r_u_*(*i*) in *s*_1_ and *s*_2_, respectively. In the CCP of *p*, the value at position *i* is the RMSD of aligning {*r*_1_(*i*-10), *r*_1_(*i*-9), … , *r*_1_(*i*), … , *r*_1_(*i *+* *10)} and {*r*_2_(*i*-10), *r*_2_(*i*-9), … , *r*_2_(*i*), … , *r*_2_(*i *+* *10)}. Only residues appearing in both protein structures are considered. Thus, disordered regions that lack atomic coordinates in either or both protein structures have null values in this profile. Structural alignment is performed using THESEUS (30), a maximum-likelihood method for superimposition and analysis of macromolecular structures. In comparison with conventional least-square methods (31, 32), THESEUS down-weights variable structural regions for a better superimposition.

## Potential binding target sites

Binding target sites are protein regions that bind its target molecules. Spatial closeness to binding targets is used as an indicator of binding. In this binary profile, position *i* is true if any heavy atoms of *i*th residue is within five angstroms to at least one heavy atom of binding targets and is false otherwise. Molecule names of binding targets that are within three angstroms to a residue are associated to that residue. Users can view this information by moving the cursor over this profile. In CCProf, binding targets are categorized into proteins, nucleic acids, ligands and ions. In the implementation of CCProf, ligands and ions were extracted from HETATM records in PDB files. The HETATM records reveal the information of small molecules, such as prosthetic groups, inhibitors and solvent molecules. The annotations of ligands and ions are obtained from the PDBsum database (33) via the identities extracted from columns 18–20 of PDB HETATM records. Water and pseudo ligands, such as selenomethionines, are excluded. Table 1 lists the pseudo ligands used in this work.

**Table 1.** List of pseudo ligands used in this work

Ligand identifier in PDB	Ligand description
LA	Lanthanum ion
LU	Lutetium ion
MSE	Selenomethionine
OS	Osmium ion
PT	Platinum ion
RE	Rhenium ion
SM	Samarium ion
SR	Strontium ion
WO4	Tungstate ion
XE	Xenon ion
YB	Ytterbium ion

## Secondary structure

Secondary structures are three-dimensional conformations of common local segments in proteins and are important for protein folding and function (34). Many protein databases, e.g. PDB, UniProt and PDBsum, provide secondary structure profiles. A distinct feature of CCProf is providing two secondary structure profiles for each protein: one before binding and the other after binding. The advantage of presenting these two profiles simultaneously is that users can quickly identify secondary structure transitions upon binding. The secondary structure of each residue is assigned according to the dictionary of protein secondary structure (DSSP), a set of physically motivated patterns for secondary structure (35). The DSSP program checks whether these patterns can be identified in hydrogen-bonded and geometrical features extracted from X-ray coordinates. Each residue is then classified into one of the following eight classes: 3/10-helix (G), *α*-helix (H), *π*-helix (I), *β*-strand (E), turn (T), isolated bridge (B), bend (S) and coil (C). The eight classes are further simplified into three commonly used classes by merging G and I into H and merging T, B and S into C. In CCProf, this profile contains two more classes. The fourth class is disordered (D), which stands for protein regions without stable tertiary structures (36). In a PDB file, SEQRES records tell the protein fragment that has been crystallized, while ATOM records indicate spatial coordinates of residues that can be recognized in the crystallization. Thus, residues appearing in SEQRES records but not in ATOM records of a PDB file are disordered. Integrating disorder information into secondary structure profile is helpful for studying disorder/order transition, which is critical to many interactions (37). Residues in the first three classes, which have explicit secondary structures, are ordered residues. Thus, users can visually recognize disorder-to-order or order-to-disorder transitions with the two secondary structure profiles. Finally, the fifth class is null (N), which stands for protein fragments that are not crystallized in PDB files. Residues appearing in the UniProt sequence of a protein but not in SEQRES records of its PDB file are classified into this class.

## Conservation

Conservation is a useful index for identifying important protein regions (38–40). Conservation profile is a real-value profile on which each position *i* indicates the evolutionary rate of the *i*th residue. Many formulas for calculating conservation have been proposed (41). But none of them performed overwhelmingly better than others (41, 42). CCProf adopts an independent-count weighting scheme combined with an entropy-based index. This combination is the most sensitive measure in the evaluation conducted by Pei and Grishin (42). The conservation values shown in CCProf have undergone an extra normalization step. The adopted conservation score is an entropy of frequencies of 20 amino acids. An entropy of 20 frequencies is in the range from −(1/20)·ln(1/20) ≈ −2.996 to 0. An entropy of −2.996 indicates the highest randomness and the lowest conservation, while an entropy of 0 indicates the highest randomness and the highest conservation. CCProf normalizes the raw conservation scores from the range of [−2.996, 0] to [0, 1] linearly.

## Database interface

**Figure 3.**
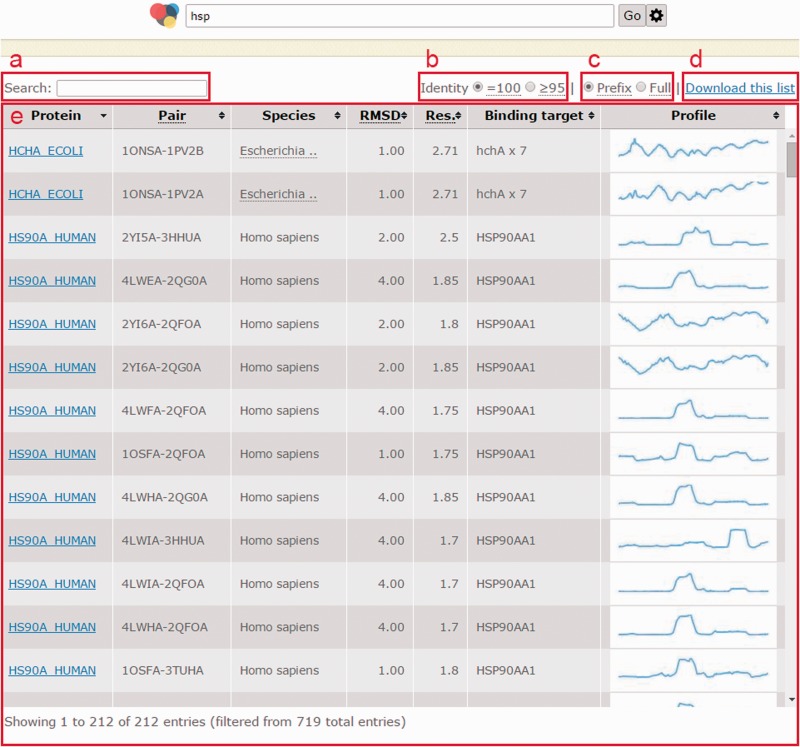
Page when a query returns multiple structure pairs. (**a**) Search field to filter current results instantly; (**b**) a switch to show pairs with identity = 100% (no substitution) or those with identity ≥95%; (**c**) a switch to turn on/off trimming long binding target description with an ellipsis; (**d**) all information shown in this list as well as intermediate data to generate this list can be downloaded with this link; (**e**) list of structural pairs satisfied users’ queries. The fields in (e) are described as follows. ‘Protein’ shows UniProt ID of the protein in that row; ‘Structure pair’ is a string of 11 characters to specify protein structure before binding (1–4 characters indicate the PDB ID and the fifth character indicates the chain ID) and after binding (7–10 characters indicate the PDB ID and the 11th character indicates the chain ID); ‘RMSD’ is the global RMSD by performing structural alignment on the two protein structures; ‘Resolution’ is the worse (i.e. larger) crystallography resolution of either PDB file; ‘Binding targets’ lists molecules appearing in the PDB file after binding but not in the PDB file before binding; ‘Profile’ is a static preview of the corresponding conformation change profile.

The home page of CCProf provides a clean and powerful search facility for exploring protein conformational changes. Users can use protein name, ligand name, domain name, ion name and even Enzyme Commission number to query CCProf. Logical operators (AND and OR) are also allowed. For more operations (e.g. to browse and to download CCProf entries), users can click the ‘cogwheel’ button. If a query returns more than one protein structure pairs, all returned pairs are listed with basic information, including protein name, PDB file, species name, global RMSD, resolution of PDB file, binding target and CCP preview (Figure 3). Global RMSD, following the same definitions of *p*, *s*_1_ and *s*_2_ in Section Conformational change, is obtained by performing structural alignment on *s_1_* and *s*_2_ according the mapping through *p*. This list can be sorted by any combination of the above fields. For example, the pair that undergoes the largest conformational changes (i.e. having the largest global RMSD) among those that have the best crystallization quality (i.e. the pair with the smallest resolution) can be identified by clicking RMSD header and then clicking resolution header with ‘Shift’ pressed. Users can specify further terms in the search field (Figure 3a) when a query returns too many results. This facility is implemented as a client-side component. This means that operations via the search field do not send any requests to sever, leading to better user experience and less server loading. All information in this list, including a text version of this list and image files for CCP previews, can be downloaded with a single click (Figure 3d).

**Figure 4.**
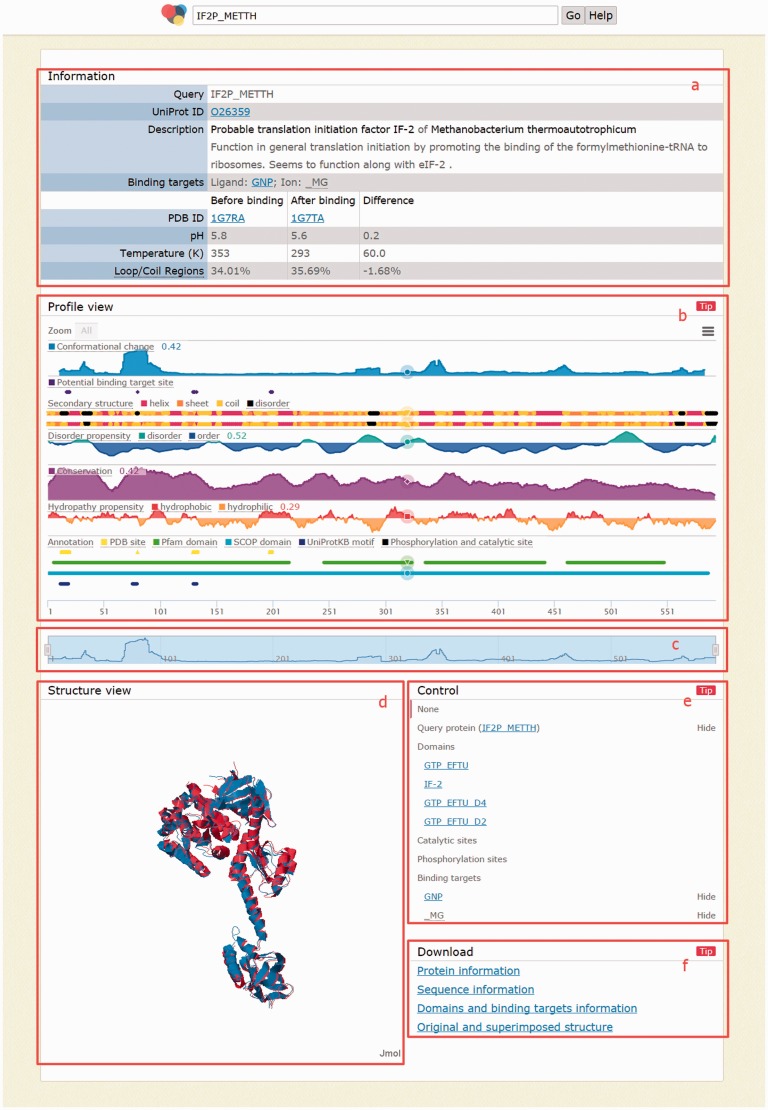
Page when a query returns exactly one result. (**a**) Information area shows the query and details of the returned protein, including the comparison before and after binding; (**b**) profile view area shows 10 biological profiles; (**c**) navigation bar for zooming/scrolling the profile view area; (**d**) structure view area shows structures before binding (in blue), after binding (in red) and binding targets (as spheres); (**e**) control area for showing/ hiding and highlighting molecules in the structure view area and (**f**) download area provides links to download the information of the above four areas.

Users can click a protein name to enter the next page (Figure 4). If a query returns only one protein structure pairs, users will reach this page directly from the home page. This page consists of five major areas. The information area (Figure 4a) shows the query and details of current protein structure pair, including UniProt ID, protein description and binding targets as well as PDB IDs, pH, temperature and percentage of loop/coil regions before and after binding. The profile view area (Figure 4b) shows 10 biological features: conformational change, potential binding target site, secondary structure, conservation, disorder propensity, hydropathy propensity, sequence domain, structural domain, phosphorylation site and catalytic site. The protein sequences appear when the number of amino acids viewed is < 160, preventing character superimposition. This area integrates all these biological features into an aligned, compact and interactive chart to make studying relevance among biological features as easy as possible. One can zoom in by simply dragging in the chart or by manipulating the navigation bar (Figure 4c). The latter provides intuitive navigational operations such as zooming in/out and horizontal scrolling. The structure view area provides a JSmol (http://wiki.jmol.org/index.php/JSmol) to help users recognize spatial relations between both states and between query protein and binding targets in a three dimensional view (Figure 4d). The profile view and structure view are linked. When a profile is clicked in the profile view, the color of the sequence in the profile view and the protein structure in the structure view change according to the intensity of the selected profile. Similar to the profile view area, the structure view area is also interactive, where users can rotate (dragging), zoom (dragging with ‘Alt’ pressed) and translate (dragging with both ‘Ctrl’ and ‘mouse right button’ pressed) molecules in real time. Users can also use the control area (Figure 4e) to show/hide or highlight any molecule in the structure view area.

Finally, all information shown in this page as well as important intermediate data for generating them can be downloaded in the download area (Figure 4f). For the information area, CCProf provides a text file containing the same information shown in this area. Next, CCProf provides the protein sequences and raw data used to plot each profile as well as the chart screenshot. For the structure view area, CCProf provides (i) both PDB files before and after binding, (ii) a synthesized PDB file containing the two PDB files after superimposition, (iii) two sequence alignments of the two protein structures against the UniProt sequence and (iv) structural alignment by THESEUS. Finally, CCProf also provides a text version for the control area, which includes domains, catalytic sites, phosphorylation sites and binding targets.

## Case study

The IF2/eIF5B is a translation initiation factor that conserves in many eukaryotes and archaebacteria (43). This monomeric G protein plays a critical role in protein synthesis (44). Roll-Mecak *et al.* (45) identified three crystallography structures for IF2/eIF5B, representing three states: free enzyme, inactive IF2/eIF5B-GDP complex and active IF2/eIF5B-GTP complex (45). The free enzyme is the state before binding; the inactive IF2/eIF5B-GDP complex is the state that is going to bind and the active IF2/eIF5B-GTP complex is the state after binding. Thus, this case study used the first protein structure (PDB chain: 1G7RA) and the third protein structure (PDB chain: 1G7TA) as the protein structure pair before and after binding and the binding target is GTP. Roll-Mecak *et al.* used nonhydrolyzable GTP analog guanosine-5′-(β,γ-imido) triphosphate (GDPNP) in the crystallization and labeled it as ‘GNP’ in 1G7T. In this context and the result page of CCProf, GTP, GDPNP and GNP indicate the same molecule.

In this case (Figure 4), the region of residues 1–225 is the G domain of IF2/eIF5B. Roll-Mecak *et al.* showed that four GTP binding motifs (G1, G2, G3 and G4, corresponding to positions 15, 80, 130 and 200 in this figure, respectively) in the G domain are highly conserved. In CCProf, these four positions clearly correlate to the potential binding target site profile (the purple profile in the figure). Furthermore, the CCP shows that G1 and G2 undergo large conformational changes upon binding GTP, while G3 and G4 do not. This observation can be explained after taking the secondary structure profile into consideration. Both G1 and G2 have disorder-to-order transitions, but G3 and G4 do not. The region near G2, which has the largest conformation change in the entire protein, is close to both the binding target and an essential cofactor, magnesium. Roll-Mecak *et al.* denoted this region Switch 2 (residues 76–94). Additionally, the secondary structure profile shows that G1 and G2 are not the only disordered regions. The region of residues 33–39, where 33, 38 and 39 undergo disorder-to-order transitions, is the longest disordered region in the G domain. Furthermore, this region undergoes a larger conformational change than G1. Based on the information shown in CCProf, one can infer that the region of residues 33–39 is highly flexible. This inference is consistent with Roll-Mecak *et al.*, who concluded that the region of residues 32–44 (Switch 1) is part of the effector region responsible for interactions with different effector proteins. Roll-Mecak *et al.* also reported that Switch 1 varies in length and sequence among G proteins. Such regions that recognize multiple targets with variable length are usually highly flexible.

The aim of this case study was to demonstrate that biological hypotheses can be easily constructed when information are integrated in a well-designed presentation. Further in-depth analyses are needed to verify these hypotheses.

## Comparison with other databases

In comparison with PSCDB, CCProf provides conformational changes for entire proteins (instead of protein regions) and covers four times more proteins. This difference comes from different strategies rather than better method. The PSCDB is a relatively accurate database, in which each entry is well studied and some preparation steps require manual intervention. This high-quality resource of conformational change is surely needed. In contrast, CCProf is a relatively automated database in which the preparation process relies on some assumptions. The CCProf scans and shows much more data and is particularly useful to study novel conformation changes. In biology, such tools for exploring new territories are also necessary. When the number of protein structure pairs is considered, CCProf has nearly 50 times more entries than that of PSCDB. This difference is owing to that one protein can have exactly one protein structure pair in PSCDB but may have multiple protein structure pairs in CCProf. The process of choosing representative protein structure pairs in PSCDB requires manual selection and lacks clear rules. In CCProf, this problem was solved by using binding targets for protein state determination. In this regard, the design of CCProf is more reasonable since one protein may have different binding targets under different conditions in a living cell.

**Figure 5.**
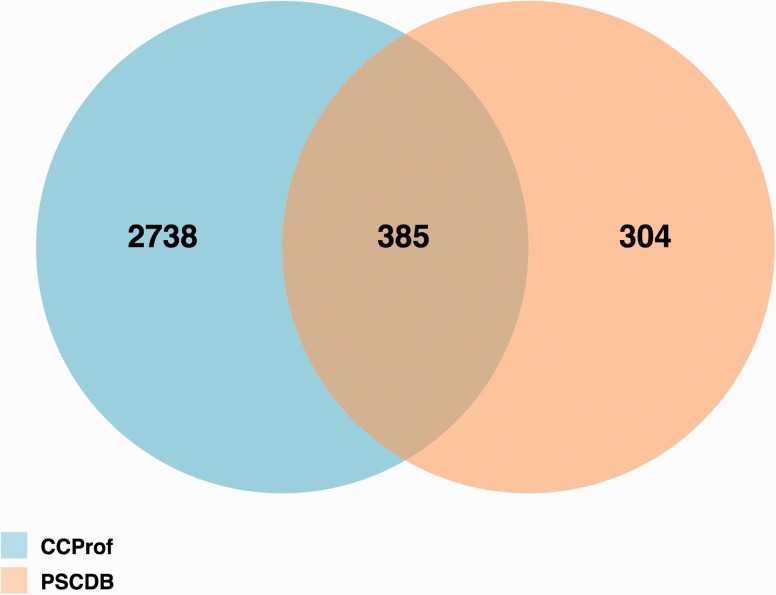
Protein overlap analysis of CCProf and PSCDB.

Figure 5 shows the results of an overlap analysis between CCProf and PSCDB. The analysis was performed at the protein level since one protein may have multiple protein structure pairs in CCProf. The complete protein lists can be found in the Supplementary Data. As a result, CCProf entries span 3123 proteins, while PSCDB entries span 689 proteins. In total, 385 out of 689 (55.9%) of PSCDB proteins are covered by CCProf. The 304 proteins were not included in CCProf because their sequence alignments did not start or end at the ends of PDB sequences. In contrast, the 2738 proteins were not included in PSCDB because the definition of ligand-free structure applied in PSCDB. In PSCDB, a structure before binding cannot have any ligand. In CCProf, a structure before binding may have ligands, as long as the paired structure covers the same ligands. In addition to entry quantity, CCProf have four advantages: (i) comprehensive CCP, (ii) potential binding targets that are generated automatically, (iii) two profiles for secondary structure and (iv) the interface to present them.

**Figure 6.**
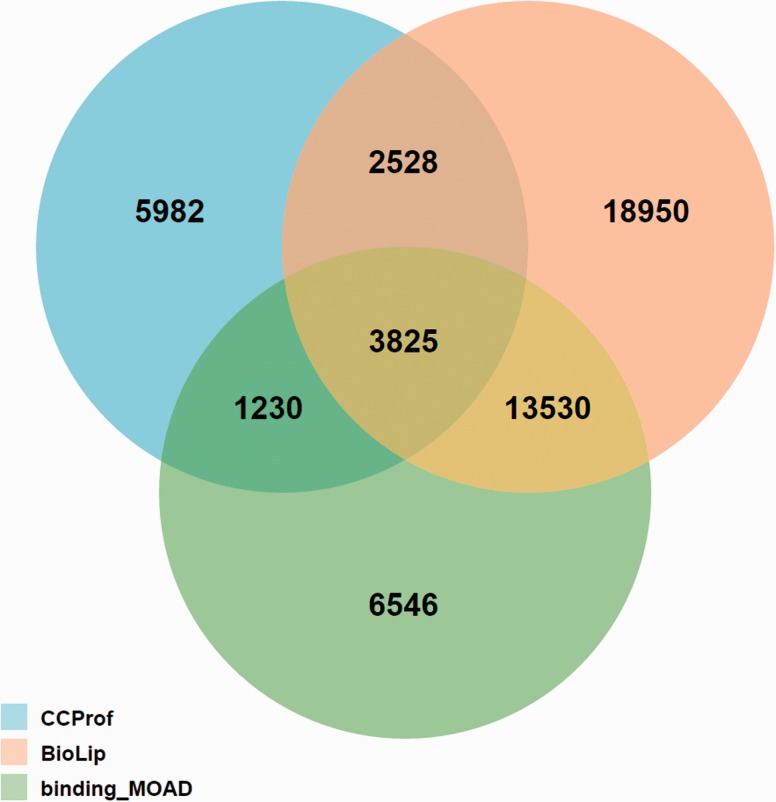
Overlap analysis of protein-ligand pairs among CCProf, BioLip and binding_MOAD.

The potential binding target sites reported by CCProf were compared with two semi-manually curated binding databases, BioLip (46) and binding_MOAD (47), for a consistency analysis. The release 7 August 2015 of BioLip contains 321 562 PDB chain-ligand pairs; the release 2013 of binding_MOAD contains 23 269 PDB chains; while CCProf contains 986 187 structure pairs before and after binding. This analysis was performed in the protein-ligand pair level. As a result, entries of BioLip, binding_MOAD and CCProf spanned 38 833, 25 131 and 13 565 protein-ligand pairs, respectively. Figure 6 shows that 7583 out of 13 565 protein-ligand pairs (55.9%) in CCProf were consistent with BioLip and/or binding_MOAD. Furthermore, the overlap between BioLip and binding_MOAD is 37.2% to their union. This small overlap reveals the difficulty of building a comprehensive database based on manual curation. In this regard, the 5982 protein-ligand pairs that were only reported by CCProf play a complementary role to known protein-ligand pairs and provide hints for further studies.

## Known limitations

One limitation is that CCProf only provides hints but not answers. Therefore, CCProf adds the term ‘potential’ before binding target site profile. To solve this problem, CCProf provides many biological features and aims to improve the analysis quality by accumulating multiple lines of evidence. But the implementation of some biological features needs further improvement. For example, binding target, which determines protein state, is in a critical position in CCProf. Adding energy calculation to assist current geometry method is an immediate next step. Another limitation of CCProf is focusing on binding target. The CCProf requires that the paired PDB files must have at least one different molecule, which is identified as a potential binding target. Though the size of CCProf is comparable to that of other databases in terms of entries and covered proteins, relaxing this requirement would enlarge CCProf to another scale and let CCProf helpful for analyzing conformational changes caused by more diverse reasons such as by temperature, pH, oligomerization state, mutations and post-translational modifications.

## Conclusion

This work presents the CCProf platform, which provides comprehensive information and a sophisticated interface for exploring conformational changes in proteins and their possible causes. All information is visualized in a unified and well-aligned manner, which is critical for capturing the relevance of different biological features. Possible applications of CCProf include analyses of protein disorder, secondary structure transition, protein flexibility/plasticity, protein interaction, post-translational modification and molecular dynamics. The update script of CCProf is executed weekly. The actual update frequency, however, depends on whether new pairs can be generated based on the updates of the source databases such as PDB and UniProt.

## Supplementary Data

Supplementary data are available at *Database* Online.

Supplementary Data
