# Supplementary material for: CCProf: exploring conformational change profile of proteins
Source: Database (Oxford). 2016 Mar 25;2016:baw029. doi: 10.1093/database/baw029 (PMC4808249; doi:10.1093/database/baw029)
Supplement: Supplementary Data [file supp_2016_baw029_index.html]

Supplementary Data 

# CCProf: exploring conformational change profile of proteins

## Supplementary Data

files

- Supplementary Data - doc file
