# Supplementary material for: CCProf: exploring conformational change profile of proteins
Source: Database (Oxford). 2016 Mar 25;2016:baw029. doi: 10.1093/database/baw029 (PMC4808249; doi:10.1093/database/baw029)
Supplement: Supplementary Data [file supp_baw029_ccprof_supplementary_data-v2.doc]

CCProf: exploring conformational change profile of proteins

Chai-Wei Chou, Che-Wei Chang and Darby Tien-Hao Chang^*^

Department of Electrical Engineering, National Cheng Kung University, Tainan, 70101, Taiwan;

* To whom correspondence should be addressed. Tel: +886-6-2757575 ext. 62421; Fax: +886-6-2345482; Email: [darby@mail.ncku.edu.tw](mailto:darby@mail.ncku.edu.tw)

Present Address: Darby Tien-Hao Chang, Department of Electrical Engineering, National Cheng Kung University, Tainan, 70101, Taiwan

## Supplementary Information

Supplementary S1 - List of proteins corresponding to the venn diagram for overlap analysis.

Contained in CCProf, but not in PSCDB (2738 proteins):

P0A7L8

P27577

P60624

P0A6T9

P00825

P02872

P12995

P00524

P04390

P73925

P01584

P29474

P06229

P42196

P62979

O31168

Q15543

O15247

P14174

Q6P2Q9

P22512

P13255

P10933

P07570

P38424

P52270

P21179

P54787

O57724

P12499

P26222

P60578

P00157

P00915

Q94524

P02931

P0A110

P09960

P20340

Q55835

Q29U70

P75792

P00508

P04253

P01138

P0A6I0

P27320

Q84G06

P03206

P0CD66

P00797

P30289

P81238

P41240

O49482

O31663

Q15466

P08476

Q03243

Q13126

Q15819

P56740

P08887

P17378

Q56310

P25098

P09486

P13804

P40859

Q99497

P16113

P00383

P03951

P15926

O77421

P06756

Q9JZ55

P50895

P56221

P07741

Q47200

P09237

P16455

P16317

P12758

O42807

P37173

Q12522

P43529

P07650

Q47454

Q9CAQ2

P32081

P00363

P38996

P13449

P19938

P28790

P0A862

P40302

P00343

Q58AD3

P19080

P0ABH9

P30074

Q01234

P02787

P07108

P05364

P03265

P23905

Q47066

O00391

P55038

P62157

Q8NKX2

P80029

O09460

P02554

P19883

P31224

P00742

Q9XSC9

P05112

P43235

P82543

P15318

P31553

P05632

P10081

Q1LCS4

Q8XL08

Q27974

P41007

P0C6X7

P20248

P03612

Q28372

P17596

A0QV09

O69315

P07328

P06876

P11405

P45202

P45796

P00451

P0ABZ6

P33176

Q08698

Q9DGB9

O33820

P08709

Q9BW91

Q9UKL6

Q8NBK3

P0A592

P0A6K3

P06210

P07477

P29549

P55915

P0A9H1

P13726

P0A9H3

P01180

Q8RQE7

P62617

P14916

Q9SWE5

P00794

P08854

A5W4F2

P06729

P26466

Q5SHP2

Q5SIY4

Q04631

P24092

P07107

P39265

P0A817

P0A0B0

P00259

P0A672

P02189

Q27686

P04964

P04695

Q07812

P00592

Q05769

P00962

P0A5Y4

O62305

Q58194

Q9DEF8

Q06187

P14193

P02761

P20226

P01901

P0A910

P0C0X8

Q57872

P51114

P00805

Q9WZC2

P04608

P14739

P11838

Q5SI28

Q16651

Q04609

Q45488

P14418

P10340

O53168

P20125

P30627

P17172

P28619

P39377

P34913

P26239

P01133

A5JTM5

P29752

P0A809

P19869

P05326

P36206

P0A7K6

Q8U1N9

P0A514

P19588

P61925

Q7SIB7

Q7M413

P00518

P0A114

Q10588

P10276

P26841

P02299

Q53H47

P03438

Q00987

P12301

P00722

P29617

P07858

P83749

P29288

P11831

P10584

P33309

Q00511

P03437

P54149

P00423

P45448

P32890

Q9ERE3

Q9IAM1

P12473

P06983

P11362

P13650

P01732

O66511

P19921

Q15303

P41016

P0A4Z6

P23254

Q58206

P61956

P00563

P15034

P85801

P00469

P05373

P03050

P00183

Q12888

P48781

P0A6E6

P16370

P18956

Q5SKN7

P24385

P76045

P09184

Q9UBT6

P13214

P41235

P10912

O60911

Q98GN8

P14756

P13319

P33150

Q9V535

Q07785

O58689

P58683

P0AEE5

O00602

O15527

P34071

P20371

P00327

P00445

Q5SGX2

P54050

P35031

Q64536

P0CG48

Q8IV48

Q9UG22

P12676

P44801

P59846

P84077

P09803

P00509

Q28021

P01887

Q05115

P0AG30

Q7M523

P06715

O59543

Q7SIC7

P62801

P0AC44

Q9Y6Y9

P10297

Q94702

Q9BY41

Q51883

P56680

Q46373

P0A0M0

Q60037

P68799

P02302

P32173

P17153

Q98157

Q8U1R4

P16048

Q0IQK9

Q56312

P24300

P25147

Q04207

P08069

Q92876

P52800

P06450

P0A9Q9

P08263

P02929

P46085

O25836

P87074

P30920

P00099

P07302

P38902

P00392

P13689

P54287

P49256

P41541

P27707

P56649

P46883

P42216

P50389

P00126

P16035

P16404

P11916

P14618

Q94464

Q52428

P18485

Q3TCN2

P07267

P27338

P54550

P69543

P15412

P00761

P00974

P13236

P02671

Q7T2Q0

P02775

P35834

P48424

P16184

P06855

P56588

P21816

P18843

Q9H2X3

P55859

P81245

Q93YN9

P30130

P16117

P0A6F1

P80302

P30291

Q15843

P15692

P04070

P22340

Q04863

P0A3C8

P69732

P61889

P42861

P02640

P07378

P00889

P0A8Q3

Q39172

P00736

P39765

P0ADY3

Q15691

P50591

P93343

P0A993

P02767

P05106

P06624

P19480

P47709

O59248

P0AC41

P80226

P22830

Q06592

Q8VZS8

Q91WR5

P61586

P96086

P04392

O00764

Q7KZF4

Q4AE70

P49012

P0C5B9

P04254

P27914

P01006

P0AEK7

P0ABQ4

P32178

P0A7M6

P12943

P27002

Q06142

O35235

P18902

P05414

P62937

P26276

P07204

P06009

P25799

Q01853

P06730

P22887

P80376

P18674

P04391

P0AB87

O60341

P14350

P07363

P17297

Q64444

P21802

Q5S007

P51698

P83798

P19711

P0A7D4

Q90VW1

P11348

O43809

Q14790

Q05871

Q9UI42

P06703

P00436

P00942

Q16665

P39956

P07996

P45148

P44688

P37954

P00734

P45974

Q9HMP7

P02263

P10175

P03195

P14743

P09788

Q42449

Q64373

P23874

P11653

P23004

P09624

O09053

P07355

P18315

P35049

Q57231

Q55080

P0A6I6

Q02763

O02372

O54709

P00448

P80099

P01215

P06911

Q03471

Q5SJH3

P00504

Q9V011

P19320

P52934

P01009

P19097

P40303

P75966

P26392

P61769

P0AG18

P29557

Q02821

Q05506

Q9Y6M4

P06026

P15056

P21243

P10760

P04936

P29466

P01903

P08836

P39462

P80030

Q9T0N8

Q53464

P14315

P10186

P0A9B2

P0C1Z0

P23009

P0AFI7

Q9BUI4

P03372

Q07841

O15151

Q57991

P30749

P01011

Q94734

P00940

P07739

P28720

Q9XJR3

P78536

P0ADZ0

P02528

Q06135

Q5SHN5

P06632

P58502

P47712

Q6GIL8

P00811

Q12933

Q50940

P0A7Q1

Q02762

Q05516

P0C2P0

Q66282

Q8BGG7

P63509

O87172

Q9H227

P56109

P78380

P84142

P21836

P01563

P49791

O75636

P0A722

Q9UBE0

P21338

Q08826

P27150

Q64537

P68261

Q5SHQ2

P00776

P07658

Q46393

Q03347

P38501

P13569

Q08506

Q71DI3

P21513

P12271

P16304

A5GZW8

P23371

Q08881

O60760

P00044

P94388

P25774

P32379

P79073

Q5SRE7

P0A8N5

P00428

P10807

P05896

P69996

P02584

Q46495

O28951

Q9UNN8

P13183

P26827

P15530

P09041

P14725

P02526

O15169

P09099

P24472

P27213

Q9DF52

P02647

P0A108

Q58987

P0C0Y9

P40189

Q9X2H9

Q6J8I9

Q9NXS2

P14336

P05089

P08202

P01308

P00125

P01236

P60775

P69202

P0A5B1

P36222

P46467

P10929

P21852

P0A8Q0

P33120

O46469

O60674

O15394

P05046

P0A7Z0

P50225

P12851

P74873

P0AB80

P06956

P30656

P02792

P00767

P12004

P0A7J0

P0A6P9

P02943

P00791

P01891

Q5SHN6

P01317

P21853

P52011

P0A786

Q9TUM0

P71447

P20345

P19560

Q96LI5

Q5SHN7

P12956

P27000

P04871

P0A855

P26661

P03962

P17424

O31440

P39075

P25651

P07030

P69905

P08337

P83792

P03042

P00484

P02240

P29603

P01751

P00727

P19619

P84079

P17743

O07529

Q9WY63

P03355

P11859

P03316

P13543

P48650

P28161

Q7LZ71

Q13485

P60460

P52197

P46680

P15569

P00939

P29460

P0C6X1

Q9WVT6

Q14974

P23951

O67648

P03621

P04166

P07471

Q57573

P13009

P80702

P14779

P13760

Q00019

O59147

Q6DE08

P35914

P26447

O81192

P76502

P0AAI9

E9Q401

P61823

P02753

P0A7A9

Q93088

Q7SIG4

P61949

P06731

O46419

P02042

Q96T66

Q01698

P00459

Q05489

P00130

P00437

P23385

Q9LDC0

P17412

Q13114

P55210

Q8MU52

P15531

P02867

Q01083

P17678

O15392

Q9FK51

P31013

Q7SIA8

Q56220

Q0QF01

P23222

P13551

P0A4W8

P03302

P06971

P07597

Q15118

P08877

P22869

Q5SGX1

P19221

P07014

P27796

P00780

P0C0Y8

Q12923

Q9WMX2

Q8RR57

Q39054

P00433

P00864

P03474

P0A5R0

P49424

O75223

P00491

Q9NWZ3

P01583

P10147

Q69014

P23360

P00214

P80912

P83795

P07845

P16098

P01070

P26790

P84092

O05701

Q9X0C7

P56734

P06681

P46798

P08046

O34919

P01233

Q9HAN9

P50578

Q9DCL8

P34752

P30274

P81186

P07288

P20435

P14831

P78330

P84051

P07371

Q10337

O66513

Q9V119

P0AES4

P0C2E9

P02468

P61326

P25685

P49053

P0A7G6

P45881

P00709

P0A5N4

P69856

P00743

P00790

P06202

P09955

P19878

P29768

P10114

P08192

P26769

P14921

P0A7K2

P28676

O88844

P33186

P30748

P61112

P29965

Q12629

P59336

Q5SHQ1

P0ACQ4

Q7LZK5

O66490

P48842

P08659

Q01043

P48728

Q56221

P56273

Q8J0D2

P25339

P09488

Q9T0I8

P02751

P60043

P23946

P15555

P0AFL3

P49228

P05631

P23921

Q65CX5

P24183

Q7M418

P09874

P63562

Q49610

O75015

Q97ZF4

P73241

P80371

Q16531

P01026

O60658

Q08603

Q5SME3

P53555

Q9EVV4

P35160

P46828

P16458

Q99P87

Q86US8

P36897

P50187

P49028

P63705

P43316

Q9S1E5

P63810

Q05315

P33681

P00883

P21953

P03472

P07464

P63280

P30153

O60603

P12319

P56689

P06129

Q5KSB7

P05164

P00875

P06628

P07986

P04233

P0AEK4

P61497

P06820

Q84KJ5

P60903

P15374

P02413

P83876

P60339

P16027

P03013

O73946

O95149

P80293

P0ABD8

P02762

P50870

A7MB62

P0ADY7

P05187

P0CG30

P01391

P04386

P04802

O96935

P25114

P42262

Q9V099

Q04416

Q89ZI2

P0A6R3

O58236

O08808

P28700

P46925

P26935

P10802

P55329

P40554

O64411

P0A6I3

P54583

P31697

P10969

P40233

P01730

P05102

P60022

P68400

P00968

P01130

O00214

P02984

P0AES0

P31243

P10153

P55931

Q09028

P20436

Q9Y337

Q9HUM0

P44539

P47068

P00378

P30405

P69786

Q51742

Q9AFI5

P02522

P62155

P15873

Q9TY95

Q13241

P0C890

Q9P0U3

P10599

P62799

P23827

P49137

Q56219

P83407

P07751

P59665

Q93VA3

P0A5S6

Q9WV48

P00326

Q9NWX6

Q9Y3B4

P07445

Q6GGU4

P11986

Q13526

P35021

Q8I914

Q13426

P0A530

P02070

P18670

P00289

P81054

P22635

P83772

P12620

P20023

P00276

P62871

P0C2S5

P13271

P16100

P00963

Q5SLV5

P49771

Q8RQE9

P07547

P0A794

P52700

P37487

P61316

P30113

P42530

P0A6Y1

P0A7L3

P09598

P80735

P19920

Q13291

P0AES2

P35030

P61320

P83791

Q02248

Q8WWM9

P02586

P80366

P0ACG8

P0A6L2

P17119

P53355

Q14691

Q9Y5Y6

P42305

Q15078

P07872

P0A6F5

Q00458

P18891

P00690

O87880

Q08831

P08997

P13981

P21874

Q9P4R4

O66949

Q28943

Q820T1

P01092

P21873

P11652

P00390

P0A9P4

P06010

P35244

P01892

P00703

P15245

P13254

P02743

P06169

P13507

P56255

P00094

P03950

Q9UIK4

P74902

P26663

Q93K97

P05630

P22619

P14859

Q43207

P04745

P55818

P10245

Q8AXY1

Q9UNL4

P36351

P22411

O34714

P05057

P17707

O34667

P96420

Q56215

P0AER0

P55135

Q07688

P00799

P0DJA2

O58320

P0A9M0

P06717

P07320

P0AG48

P03034

P34097

P51649

P62158

P0A4Z2

Q38087

P47244

P10282

O58035

Q6B856

O66186

P11204

P15214

Q969H0

P11177

P0A6X7

P12904

Q60099

O25776

P25451

P13134

Q57DY1

Q57849

P02745

P07662

P24182

P35804

P04040

P59666

P0AGE9

P10600

P68530

O95150

P00693

P77072

P05161

P15319

P18676

Q67333

O34777

P63228

P63248

Q9GZT9

P0A7C2

P40363

P00503

O25896

P04075

Q16658

P22307

P01034

P02974

P0AD64

P65477

Q07982

P02283

P20425

Q5SHN3

P08536

P00711

P53365

P02836

Q59990

P29717

P0A9B6

P42574

P80958

P02747

P86041

P05455

P00750

P11349

O75151

Q27709

P00829

P11562

P0AE12

Q96LB9

Q56839

P04252

P14649

P41391

P45697

P0AFI2

P02766

P08179

P67700

Q86WA6

O74213

P0A6W9

P30685

P01625

P04552

Q16875

P00654

P18798

O69250

P35222

Q28175

P18910

P22303

P0A9J4

D0VWV4

P0A7N9

P06654

Q13231

P15587

P15917

Q56148

P37967

P19999

O33819

O32210

P01542

P02924

P01966

P00480

P11717

O58389

Q59643

P00752

P0C6E9

Q9H6P5

P0AC47

A5W4F1

P03300

P14775

P21397

P26789

Q05599

P00280

Q9SE42

Q8J136

O15519

Q9GK12

O66188

P00270

P01241

P29894

P00747

P15167

P08107

P81459

Q7M419

P13482

P22139

P0A5Z8

Q9X519

P00381

P00426

O15540

P22392

P49366

P0CH62

P38636

Q9P4R8

P00429

Q29460

O08967

P03069

Q9C1S9

P0A738

P06609

P43889

P17599

Q8NHL6

Q02242

P18177

Q65XK0

P34170

P06786

Q96FQ6

P0C6U2

P67870

P46672

Q01786

P20783

P42345

P02699

P0A6Z6

P41052

P19812

P03958

P68807

Q9X286

P06611

Q91132

P60438

Q9GZX6

P20586

P20933

P49435

P0AF28

P00171

P45452

Q01693

P34629

P04587

P19367

P0AFK9

P13272

O77811

P25440

Q9XDH5

P25454

P07470

P57736

Q02169

P44542

P0A6Z3

P29899

Q58584

O74866

P04531

P24627

P40202

O16025

P0A884

P00749

P07688

P0ACS2

P23577

P36217

P67876

P0ACC7

P04825

P60338

Q8RQE8

P21673

Q9S400

P56252

Q9Y8H8

Q8WXF7

O29912

P68246

P28313

P80721

P68135

P00766

P29715

O67728

P13127

P05362

P20936

O66496

Q9FA38

P17998

P0C5C1

P84022

P43876

P0A7N4

P18187

O28323

P03495

P07329

P62877

Q5SJ28

P18000

P11987

P68919

P25984

P33284

Q56218

O66529

Q8SR45

P09186

Q00459

P18188

Q27743

P0A9J6

P18203

Q15369

P24058

O15382

P19400

P58301

P36649

P07798

P08839

P0A2D5

Q3SYX9

O06162

P03303

P0A853

P17571

P06127

P14941

Q4GWU5

P40149

P61503

P68826

P84233

P02633

Q9LV40

P39593

P15018

P66799

P0A6V5

P03322

P08833

Q00457

P38038

P22535

P02511

P03276

P0ACS5

P70994

Q61823

P21793

P38940

P0A8I1

P35247

P23869

P23847

P07830

Q97W02

P02208

P29600

P09382

P00323

O33832

Q07960

Q007T0

P12282

P21146

P60723

P07649

P56218

P35755

Q9X0I0

P00512

P48061

P19984

P39900

Q5SLQ0

O70351

P04418

P03366

P69488

Q00022

P68871

P19157

P0C0R7

P04584

Q9R8E3

P62149

P0A0Z8

P07801

P10958

P20434

P05725

Q8U160

P11941

P00777

P0CE47

P00698

P16094

P63709

Q3T035

P0A9T0

P02675

P84140

Q8TG90

P25080

P02994

Q03023

P0C918

P00492

P80457

P22748

P0A6C8

P12257

Q05097

P71119

P31371

Q5SJ80

Q14749

Q9RQB9

P24941

P19515

P13987

Q9NPB1

P18075

P80372

P08692

Q5SKZ7

Q58235

P69783

Q9L5D6

Q2FV22

P00763

P04036

P06400

P03051

P04425

P04190

P62837

Q9Y279

P80424

P28147

P39621

P80025

Q9XZT6

P49789

P43092

Q46455

Q01705

P29350

P04574

P65716

O69639

P31723

P07291

P02911

P03306

Q819U0

O02604

Q01594

Q9RS64

P19793

P12944

P23370

P17676

Q59560

P10408

Q84FH6

P26231

Q9D967

P23882

P08515

P23457

P0AGJ9

Q10740

P05981

O60880

P11961

P0A6F3

P05618

P00787

P25491

P27001

P0A7M9

P0A800

O75533

P40422

P61972

P30986

Q00534

P10478

Q56WD9

P68082

O73948

P80401

Q5SIH3

Q13541

Q16539

P54300

P00952

O28751

Q9R5V5

P23639

P20906

O58677

P29477

P53051

O06644

P13299

P06213

P0CE48

P02213

P02794

P34884

P21457

Q9I4V0

P00639

P07686

P96142

P07062

Q24117

P43214

P41958

P10145

P00519

P0C188

P00431

O13024

P12281

P07463

P15363

P05042

P07620

P50224

Q5SJ76

P10144

Q977P5

P46422

Q47898

P69910

P07824

P0A825

P03018

P62825

O02697

P06873

P01556

P37554

Q14289

P09147

O66646

Q9S7E4

P00959

Q60176

P0AAJ3

Q04432

P00325

Q58761

P47205

P0ACJ0

O32462

Q02761

P34087

Q9WYW0

Q53654

Q05397

P46859

P47228

P35658

O43252

Q92888

P0C0I6

P65865

Q7T2Q1

P03882

P02062

P52293

P00808

Q6P6M7

Q7NSA6

P55273

P0A7Z4

P0C1A2

Q9SE93

Q03708

Q56224

P05769

O67757

P02774

P56658

P21524

P0A7F3

P37698

Q9KM65

O43566

P90551

P07132

P01575

P37062

P11456

P30481

P91938

P19972

P17315

P0AFJ5

P54763

Q43866

P36924

P40136

Q9A5I0

Q15418

P29563

P38051

P00775

P61926

P00573

Q13616

Q56404

Q5SLP1

P50910

P0A574

P01958

P48391

P34697

Q7SID3

P0ABJ1

Q9WX76

P00807

P22301

P56199

P28504

P0AG44

P17846

P00430

P23807

P83797

P27988

P56216

P62826

P45206

P22364

P29283

P52663

P20083

P0A7R1

P54760

P01053

P02930

P11310

Q84424

P01112

Q14676

P05019

P15445

Q05514

P08427

P00442

O66037

P46072

P39662

P80078

Q14995

P00517

P04637

P08164

O32449

P25553

P03521

O00092

Q10651

P0AA04

P69986

Q92871

P80188

Q9X5C9

P54322

P81122

P10275

Q56222

P84040

P0C018

P05045

Q6ZMT4

Q1XA76

P01087

P49841

P80385

Q8ZKF6

P16384

P79345

P0A805

P02693

P33221

P03989

P07511

P05543

Q5SIA8

O06143

Q99714

P01493

P60422

Q9NVD7

Q15796

Q16773

P10104

P54965

Q9UN36

Q5SM60

P11157

O14757

P37330

P03956

P22894

P29396

P02789

Q51507

P16083

P04072

O09345

P0CB51

Q08751

P10056

P20433

P07173

Q495M9

Q9UK55

P12931

P80276

P04050

P28324

P0A759

P06746

P00778

Q48255

Q15596

Q7Z4W1

P84080

P00947

P0AAI5

P10880

P81446

Q9KWU8

P80377

P00175

Q5SLR4

P31808

P81637

Q06520

P39958

O58456

P12807

Q70626

P24295

P38489

P62576

P84887

P01024

P03452

P76469

P98170

P84612

P39315

Q08753

P20701

P19919

P0AGE0

O50008

Q9RXJ5

O80992

Q50497

O93655

Q58108

P41367

O26232

P80380

P02746

O33840

P29476

P48026

P37063

Q07889

P71039

P32639

O32221

P24474

P36275

P17931

Q5SHR6

Q04894

P10902

Q9ZMY2

P05452

P27986

P84229

P0A3C7

P0ABH7

Q5AU62

P52799

P0ACP7

P53571

P08758

P11716

P01175

Q55075

P21242

O50385

Q15370

Q16654

Q03048

P17291

Q59196

Q39255

P05327

Q57679

P30043

P61175

P01031

P75914

Q8LAS7

P0A5I4

P30305

P80378

P37064

P80484

Q7LWY0

P04905

Q5HFG7

P13010

Q9ZF99

P00760

Q9D0P5

P56634

P13448

P69791

Q8RQP5

P07170

O67610

P62965

P29957

P47863

Q04299

Q9UHY7

P03775

P47229

P22141

P69795

P0A8G6

P16233

P0A7Q6

P00593

P37595

O66187

P11766

P00704

P56153

P23837

O08349

P43099

Q53239

O30126

P23472

Q9Y5S9

P15116

P13744

P22637

P20339

P26163

Q46372

P09029

P47075

P00490

P24605

P02679

Q01469

O58843

P00636

P22629

P30996

Q03181

Q00017

Q9XY07

P99999

Q57817

O58764

P22985

P34130

Q9X0C8

P14532

P0A334

Q9UUB1

P26697

P84090

P01555

P46109

P0A0L2

P13129

P20058

P53396

P21399

P07711

O34559

Q00796

Q99828

P07552

Q01196

P35729

Q92947

P60479

O74933

P08518

P42013

P04084

P0AEG6

P00582

P06875

P61914

O05793

P15636

O04986

P01012

P0ACC1

Q9KIZ4

Q5SJP8

P56968

O88522

P0ABP8

P37146

P32169

P16525

P0C558

Q972I2

P32179

P16544

P60355

P63096

P25524

Q5SHP7

P49302

P38830

P05132

P08254

P0A7I7

Q58440

Q570C0

P07617

Q8NXI6

P28867

P95468

P0ACB2

O34757

P01051

P10868

P02853

P16088

O14727

P00446

P62399

O30298

P06737

P83793

P13500

O05783

P13479

P38919

Q9UMR2

Q13153

P28631

Q01852

P27989

P83796

P03275

Q15544

P40347

Q58CQ2

Q9ES89

P11540

Q9Y4D1

P25311

P37019

P00523

P0A8V6

P36946

P17413

Q7NDN8

P09601

P07360

P96112

P03313

Q53W92

Q9RX88

P63043

P13376

P18314

P26718

Q7SIB1

P43379

P31742

P30131

P10987

P10828

P23694

P10507

Q9XG54

P15369

Q5A3V6

P25220

P56065

P15559

P03180

P02247

P02877

P15927

P05305

P60546

Q16769

O28126

P39825

P49121

Q62226

Q00277

P17109

Q99584

P62554

Q9UKK6

Q9FUP0

Q148J6

P15273

P69054

P16444

P28650

P03369

Q9I596

Q92731

P08954

O58316

Q9WZC3

P0C0V0

P00004

O05268

P11560

Q9UNA4

P00260

O43447

P27907

Q9QYY9

P22073

C0SPA0

Q9UKL0

P09211

Q992I2

P21553

P71086

Q62997

P50477

P01854

Q0TV31

P26602

Q96F86

Q8DR59

P42321

P00598

P23526

Q5SJA1

P00362

P80857

P26137

P42790

P68390

P14755

P48499

Q83884

P98066

P04179

P49765

P61157

P03692

P14668

P07998

P30617

P26393

P15093

Q923J1

P0A1J1

P56533

O81223

P09787

Q969G6

P43772

P02722

Q9KEI9

P30803

O57385

Q09161

Q9NR28

P22906

P16152

P96851

Q07108

Q5SLP8

P29395

P60896

P04278

P69179

P15452

P00299

P04117

O59791

P17989

Q96C86

P11353

P20480

P37610

Q9X183

P08246

P59594

P96382

P0A790

P0A6R0

P24146

A6XNC6

P60526

P49638

P08253

P23940

P0A5J4

P12335

P04377

Q40577

P45850

P03680

P03000

P36238

Q52675

P00700

P03700

P49914

P08311

P07254

P80373

P80040

P00129

P06276

Q99418

P06239

P20261

P0A935

Q09064

P52045

O06632

P11904

Q02293

Q9WYJ7

P0A836

P40399

P61875

P74903

P30086

P0C278

P47989

P22030

P17493

O05207

Q9RXJ7

P25043

P60523

P12823

P00549

P17888

P28793

P03012

P04122

P16581

O73947

P60044

P09681

Q75I93

P62161

Q5SJH5

P20000

O93715

P0A1F6

P21310

Q14653

P00415

P48425

P0CI76

P02925

P0A955

P08716

P23657

Q9UBT2

Q56403

P0C2S3

P0AG51

P45741

P0AEG4

Q6NS38

O05891

P15289

Q46079

P13000

P00132

P07273

P00772

O07746

P0ACJ8

P04042

P01055

P0AD61

Q7SID2

P00388

P69544

Q9V1G0

P01275

P0AG16

P33650

P38505

P12643

P03952

Q14376

Q00496

P05674

O58720

O35031

P26039

O94753

O75582

P06008

P39116

P0AA10

Q9A6Q5

P23295

P04963

P08882

P0A7P5

Q9N1E2

P39040

O33818

P63316

P0ACF0

P13340

P30657

P08191

P13696

Q8EE30

P07274

P00740

P64767

P00497

Q9I4K6

P08621

Q5SHQ5

P16703

Q9NZD4

Q3UZZ6

P41365

P19120

Q5SID2

P0A2K1

P56839

Q39761

P05230

P0A7B1

P46922

Q90249

Q63537

Q53940

O55013

O29883

P36006

P02625

P23339

P0A3Y5

P09546

Q77E03

P00138

P04629

P69681

P60293

P40881

P09012

Q02054

P20160

P0C186

Q6B2C0

P13928

P0CL52

P61615

P03040

P40482

Q27701

Q8E565

P08581

P0C1H5

P12369

O92972

P02638

P08870

P15160

P23532

P14920

P02701

P02920

P22636

Q64288

P0A6C1

P00131

P01050

Q56232

Q08499

Q62230

P60327

P15266

P06241

P07636

P01315

P24630

P17893

Q16775

Q27793

Q02745

P80645

P0C0Y7

P28630

P0A749

P24476

P03176

P53779

P00957

P05979

P03367

P0ADZ4

Q8U0R7

P0A877

Q966X9

O34153

P04746

P10943

Q07599

P08169

P11350

Q980Q4

Q14019

P15309

P12277

P00514

P07339

P25823

P31800

Q9FDN7

O60671

Q7LZK8

P13398

Q00535

P20459

O43070

P39928

Q5HJD7

Q08188

P81445

P32816

P04415

P0CI79

Q4D3W2

P01857

Q6TEC1

P00800

Q12390

P77444

P24297

P16099

P00488

Q60053

P54654

Q11176

P0C2X0

P08160

P80374

P02973

P31939

P14385

P07591

Q40778

P10775

P12694

P04271

P03023

P12268

P03001

O50580

P21980

O75496

P61517

Q99728

O59580

P00489

P42330

P12544

P14768

P59071

P62330

P02779

O76036

P36655

P0A434

P02945

P06672

Q8NBP7

P08165

Q44185

P09850

P52298

P60045

P52917

Q9RVD6

P05449

P38624

P15043

Q07807

P25500

P53608

P36873

O06594

P41222

Q26998

P05798

P0AG55

P08661

P25974

P23724

Q02127

P00735

O57823

P0A7D7

P02791

P23247

P17802

P0AE22

Q00456

P10121

Q977U7

P21888

P0AG82

P02550

P38117

Q3MHR7

P50097

Q53ZE5

P50163

P37821

P27142

P00396

P09527

P0A6H5

Q56313

P06897

P35228

P01270

P45066

Q01745

O59425

P16790

P23909

P08185

P20290

P28523

Q50642

Q9UKK9

P06886

Q13418

P63488

P60472

P00560

O76242

P33643

P22337

O27818

P78543

Q16611

P77541

P11759

P09152

P23638

P0A9M5

P02281

Q96323

P0A3F4

P24733

Q9YIC2

O80164

P11064

P83794

P22641

P33590

P56220

P02619

A0R3D3

P37840

P12070

Q9UBU9

P09945

P36894

P0A6D3

P00929

Q80Z29

P62152

P31101

Q97R46

Q56223

P68802

P42700

Q8L5C6

P18013

P07987

P0A4Y8

P22069

Q20728

P80561

P53570

P80176

P41407

Q9BRT9

Q9KNC3

Q7T1K6

P19483

Q54450

P07773

P29160

P04063

P00751

P22029

P00432

Q54944

O07615

Q72EC8

P52732

P0A5Y6

O60563

O15234

Q02567

Q99638

Q02899

P22487

P09391

P66032

P02357

P56210

P32055

Q05086

P0AEJ6

Q9WYN2

P23721

P53041

P39042

P56122

P21890

P12528

P31751

Q47112

P0AG74

P25401

P0A112

P05121

Q46604

P01375

P13423

Q5SLR3

Q06486

P20372

Q831W7

P0C1B3

P42419

P31749

Q4JB80

P05451

Q60365

P21963

P28314

P10824

P12306

P10515

P03315

P62593

P08174

P27999

P27038

P22643

A5U2B7

Q82122

P84888

P03036

P00798

P08631

P09872

P10997

P0A6H1

P05020

Q07412

P83223

P00862

Q00460

P18316

P34024

P04038

P0A715

Q55012

Q14671

Both contained in CCProf and PSCDB (385 proteins):

A1L259

A3DC29

O00182

O00244

O00299

O06553

O07347

O26359

O27564

O30245

O33833

O35744

O43001

O55012

O57413

O57980

O58107

O58441

O59493

O59952

O60729

O67082

O67618

O74017

O74237

O75164

O75340

O85465

O92956

O95166

O95786

P00257

P00268

P00273

P00349

P00374

P00439

P00441

P00533

P00558

P00586

P00588

P00590

P00634

P00641

P00644

P00648

P00651

P00669

P00717

P00718

P00720

P00730

P00746

P00782

P00784

P00803

P00817

P00918

P00924

P00949

P00953

P01008

P01139

P01552

P02185

P02340

P02417

P02588

P02618

P02754

P02768

P02788

P02829

P02879

P02883

P04058

P04067

P04395

P04547

P04585

P04718

P04789

P04818

P05067

P05655

P06132

P06278

P06396

P06612

P06709

P06744

P06762

P06766

P06988

P07024

P07071

P07102

P07359

P07584

P07737

P07788

P07900

P08017

P08170

P08200

P08519

P09167

P09331

P09373

P0A006

P0A077

P0A2M9

P0A3D9

P0A3E0

P0A3R9

P0A6A8

P0A6Q3

P0A7Y4

P0A881

P0A8M3

P0A8N3

P0A8U6

P0A917

P0A953

P0A988

P0A9E0

P0A9G8

P0AA25

P0AB71

P0ABE7

P0AC81

P0ACT4

P0AD96

P0AE18

P0AE67

P0AEB2

P0AEC3

P0AEX9

P0AF18

P0AFC0

P0AFG8

P0AGD3

P0C6U8

P0CH28

P10538

P10822

P10844

P10845

P11073

P11116

P11124

P11172

P11215

P11411

P11532

P11546

P11889

P11926

P12259

P12295

P12376

P12497

P12724

P12998

P13036

P13280

P13298

P13501

P13513

P13717

P13956

P14061

P14210

P14262

P14489

P14555

P14677

P14751

P14769

P14900

P14925

P15057

P15121

P15132

P15454

P15570

P15879

P15925

P16622

P16753

P17169

P17255

P17865

P17870

P17900

P17978

P18031

P18429

P18548

P19267

P19656

P19821

P20974

P21163

P22259

P22266

P22734

P22983

P23007

P23202

P23381

P23687

P24670

P24931

P25910

P26043

P26281

P26514

P27487

P27695

P27838

P28012

P28366

P28907

P29218

P29241

P29320

P29373

P30044

P30122

P30340

P30967

P31116

P31151

P31570

P31658

P31696

P31947

P31992

P32099

P32171

P32324

P32396

P32449

P32455

P33160

P33247

P33331

P33673

P34174

P34914

P34945

P35555

P36419

P36929

P37231

P37344

P37957

P38398

P39045

P39172

P40191

P40871

P42212

P43490

P44583

P44859

P44868

P45568

P46881

P47929

P47934

P48632

P49235

P49773

P50384

P50440

P51541

P52704

P52754

P52948

P53350

P54274

P54512

P54939

P54997

P56690

P56817

P56868

P56965

P59655

P60568

P61626

P61964

P62508

P62942

P62993

P68175

P68638

P69441

P69924

P77173

P77214

P81180

P82197

P83194

P83304

P83790

P96084

Q00955

Q02834

Q03161

Q05581

Q05603

Q08169

Q0TR53

Q13093

Q14203

Q15119

Q16836

Q29495

Q29551

Q2RSB2

Q46893

Q52440

Q53547

Q53591

Q53728

Q54468

Q54873

Q55891

Q56F26

Q57816

Q58989

Q58MU6

Q5L1E2

Q5SH23

Q5SHZ3

Q5SJF8

Q5SJP7

Q5SLQ3

Q63K37

Q658P3

Q6WY08

Q72J47

Q84BQ9

Q8AVA3

Q8WTS6

Q8WZM3

Q92973

Q93009

Q96EK6

Q96T88

Q980A5

Q99003

Q9CCZ4

Q9H2H8

Q9HKT1

Q9HUL9

Q9JWM8

Q9L9D7

Q9NWT6

Q9NZD2

Q9P4V2

Q9QWJ9

Q9QZ85

Q9R4E4

Q9RC92

Q9TVW2

Q9U2M7

Q9UJY5

Q9UZ14

Q9WY54

Q9WYA3

Q9WZQ4

Q9X0C6

Q9X0X3

Q9Y233

Q9Y286

Q9Y376

Q9Y468

Q9Y5X1

Q9YBQ2

Q9ZP19

Contained in PSCDB, but not in CCProf (304 proteins):

P52565

P43912

Q96C23

P9WFF5

O31662

P22027

P31044

P30419

P76216

P13835

O00834

P38503

Q8ZWV0

Q9DBG9

P27115

P0AFZ3

P21638

O15530

O94267

P13702

O15194

O13437

P15442

P9WFX5

P55984

Q9UHL0

Q9HKL4

Q8L8B8

P04995

P11797

Q6N5P6

P37798

P17778

Q92900

Q6GFF9

Q8WUM4

Q9JLU4

Q72H90

Q61830

Q6SLM1

Q9Y3R4

P32056

P00282

P23685

P54278

Q46822

Q9BRR9

Q72X44

O34928

Q60364

P17312

P48845

P26465

P14565

O00213

P23540

P60493

Q8VHN2

P0AGC3

O22265

P0A4L7

Q9UHX3

Q8ZNR3

P15379

P0DJL7

Q14558

Q8DLI5

Q02046

O34974

O86309

P12530

Q9JI78

P0DM80

P80667

P19478

P21674

Q9UUN9

P49327

P26660

Q9EY50

P97465

Q99X56

Q99QV3

P9WID5

Q9WY43

Q980M3

P39008

Q70DK5

P07813

Q60034

P56073

P9WIL5

P51452

P84155

P23904

P29468

Q9Z4Z5

Q14191

P0C1A8

P23677

Q9BY32

P9WNE1

P9WKD7

P08195

Q5HE12

Q9UPN6

P9WNV3

Q9Z2X8

O29370

P0C6Y1

O25096

Q9NUW8

O00560

P0A6V9

P21856

Q96IV0

Q58663

P0CG50

P12955

P14324

P08799

P21695

Q9VHA0

P62374

Q7UAV7

P19446

P0A8P1

P07774

O30207

P38998

P70826

Q93ZN9

O28608

Q9KU37

P00452

Q06121

Q8SR66

P9WJJ7

P0A0B9

P9WPY5

P25539

Q9QWW1

Q9UM07

O35795

P21454

Q00855

P67825

O31474

O32157

P9WGP5

P18886

P59676

Q8FBQ3

P0ADA1

P10688

O09008

P10384

P34152

Q8Y6I2

P56427

P08037

Q7A827

P37028

Q97UA0

Q96GD0

Q7MUW6

Q4U331

Q62696

P03314

Q72H91

Q8GCH2

P23901

P63005

W3VKA4

P9WQN9

O68014

P04517

P39081

Q99KQ4

P86294

P65248

Q9UBQ7

P01851

P63224

P75736

P17452

P50053

Q9X2H6

P41212

Q15257

P07953

Q13838

Q9I1X7

Q54727

P61149

P28522

O06961

Q8PWY1

Q13257

P29029

P0C6V2

P22102

P9WPF1

Q9Y5P4

P9WIC1

Q9K706

P75430

P9WPB7

Q55460

P44492

Q969R5

P0AEY5

P26446

P01709

P68568

P04958

P46934

Q58462

P50232

P16442

P76344

Q6JP77

P04015

P76015

Q6PQJ9

P44490

Q59675

P31572

P07435

O67323

P56194

P9WIB9

Q06287

P9WPY3

P07250

P27616

P96688

O75891

Q9Y4K3

P22121

Q02790

Q7A1N5

P37672

Q9TQS6

Q04724

Q8KHZ8

P22414

Q57977

P29208

Q03629

Q9HWC1

Q56320

P77425

P10968

P23833

Q58504

P0AEY7

P78504

Q6U7R4

P77933

P17427

P55882

Q55169

P0A752

Q8U3L0

Q9M9P3

Q96I25

P53582

P13727

Q99704

P07738

A9JQL9

O66145

P98052

Q9Y2R2

P46844

P48736

P9WNG5

O15389

P59636

Q9D020

Q9Y2K7

P20703

P0CS93

Q9BYN0

Q04081

Q8NFD5

P9WNG3

P40479

P0A915

P05090

Q1R415

P84801

P45523

P10246

P27652

Q9NRN7

P0C1A9

Q12149

Q09LY5

P17728

Q46389

Q9X0D0

Q9H1R2
